# Integrated health and social care evaluation framework for mental health and drug addiction care: Gabriela Barbaglia

**DOI:** 10.1093/eurpub/ckac130.115

**Published:** 2022-10-25

**Authors:** G Barbaglia, N Robles, P Hilarión, M Torres, M Gotsens, E Colell, E Puigdomènech, J Arias de la Torre, M Espallargues

**Affiliations:** 1 Universitat Pompeu Fabra, Barcelona, Spain; 2 Agència de Salut Publica de Barcelona, Barcelona, Spain; 3 Red Investigación en Atención de Adicciones, Spain; 4 Agència de Qualitat i Avaluació de Catalunya, Catalunya, Spain; 5 eHealth Center, Universitat Oberta de Catalunya, Catalunya, Spain; 6 Consorci Sanitari de Barcelona, Barcelona, Spain; 7 Fundación Avedis Donabedian, Barcelona, Spain; 8 King's College, London, UK; 9 Universidad de León, León, Spain

## Abstract

**Background:**

Guiding the decision-making process in mental health investments is advisable. The objective of the study is to develop a framework for evaluating the quality of integrated health and social care in Mental Health and Drug Addiction (MH&DA)

**Methods:**

A literature review helped to establish a definition of integrated care specific to MH&DA and to identify potential indicators for its evaluation. The quality of integrated care was assessed through focus groups (FGs) and interviews (INs) with three different profiles: professionals (2FGs & 2 INs), patients (3 FGs & 2 INs) and families/carers (2FGs & 2 INs). Additional indicators were also obtained from them.

**Results:**

Out of 2,226 publications identified, 87 (4%) were reviewed in full. According to the literature, integrated care in MH&DA is based on four main components: case management, comprehensive assessment, individualised care plan and care coordination among different providers. Based on these components, an operational definition of integrated care was developed and validated in the FGs and INs. Positive aspects identified were a respectful approach and positive experiences of coordination between social and community network. Regarding indicators about 400 were identified, after screening were reduced to 60: 25% corresponded to accessibility, 20% person-centred care, 16% each to care coordination and to effectiveness. In general, the main threats to the quality of care, identified in FGs and INs, matched the dimensions with the highest proportion of indicators (i.e., limited care resources, poor coordination and communication among professionals and services, and barriers in accessing specialized treatment).

**Conclusions:**

According to literature, integrated care in MH&DA seems to be mainly evaluated in terms of accessibility and person-centred care. In a following phase, a large group of experts will be key to select the most relevant dimensions and indicators for the evaluation in a Delphi study.

**Key messages:**

- A quality integration of Health and Social Care is paramount for people with mental disorders and/or drug addictions.
- A framework for assessing the quality of integrated Health and Social Care in Mental Health and Addictions would help guide necessary investments in mental health.

